# The ratio of maximal handgrip force and maximal cycloergometry power as a diagnostic tool to screen for metabolic myopathies

**DOI:** 10.1038/s41598-020-65797-1

**Published:** 2020-06-01

**Authors:** Jean-Baptiste Noury, Fabien Zagnoli, François Petit, Cédric Le Maréchal, Pascale Marcorelles, Fabrice Rannou

**Affiliations:** 1 Neurology Department, Neuromuscular Center, CHRU Cavale Blanche, Brest, F-29609 France; 2 Molecular Genetics Department, APHP - GH Antoine Béclère, Paris, F-92140 France; 3 Univ Brest, Inserm, EFS, UMR 1078, GGB, F-29200 Brest, France; 4 Pathology Department-EA 4685 LNB, Neuromuscular Center, CHRU Morvan, Brest, F-29609 France; 5 Department of Sport Medicine and Functional Explorations-CRNH Auvergne, Clermont-Ferrand University Hospital, G. Montpied Hospital, Clermont-Ferrand, F-63000 France

**Keywords:** Laboratory techniques and procedures, Neuromuscular disease

## Abstract

Metabolic myopathies comprise a diverse group of inborn errors of intermediary metabolism affecting skeletal muscle, and often present clinically as an inability to perform normal exercise. Our aim was to use the maximal mechanical performances achieved during two functional tests, isometric handgrip test and cycloergometer, to identify metabolic myopathies among patients consulting for exercise-induced myalgia. Eighty-three patients with exercise-induced myalgia and intolerance were evaluated, with twenty-three of them having a metabolic myopathy (McArdle, n = 9; complete myoadenylate deaminase deficiency, n = 10; respiratory chain deficiency, n = 4) and sixty patients with non-metabolic myalgia. In all patients, maximal power (MP) was determined during a progressive exercise test on a cycloergometer and maximal voluntary contraction force (MVC) was assessed using a handgrip dynamometer. The ratio between percent-predicted values for MVC and MP was calculated for each subject (MVC%pred:MP%pred ratio). In patients with metabolic myopathy, the MVC%pred:MP%pred ratio was significantly higher compared to non-metabolic myalgia (1.54 ± 0.62 vs. 0.92 ± 0.25; p < 0.0001). ROC analysis of MVC%pred:MP%pred ratio showed AUC of 0.843 (0.758–0.927, 95% CI) for differentiating metabolic myopathies against non-metabolic myalgia. The optimum cutoff was taken as 1.30 (se = 69.6%, sp = 96.7%), with a corresponding diagnostic odd ratio of 66.3 (12.5–350.7, 95% CI). For a pretest probability of 15% in our tertiary reference center, the posttest probability for metabolic myopathy is 78.6% when MVC%pred:MP%pred ratio is above 1.3. In conclusion, the MVC%pred:MP%pred ratio is appropriate as a screening test to distinguish metabolic myopathies from non-metabolic myalgia.

## Introduction

Metabolic myopathies constitute a group of heterogeneous disorders that interfere with generation of ATP in skeletal muscle. Since ATP production is several-fold increased during muscle contraction^1^, exercise tests are often part of the patient evaluation if a metabolic myopathy is suspected^2–5^. The two main functional tests are the isometric handgrip test and the cardio-pulmonary exercise test. Isometric handgrip test consists of, first, determining the maximal voluntary contraction force (MVC) of muscles from one forearm, and, secondly, performing a 30-s contraction at 70% MVC^2,3,5^. Cardio-pulmonary exercise test involves dynamic contraction of lower limbs on a cycloergometer, and employed a progressively increasing workload until maximal power (MP) is achieved^4^. These two functional tests aim to unmask a metabolic defect by measuring muscle metabolite production from blood samples following a standardized ‘laboratory’ exercise^2–5^. Lack of exercise-induced increase for a given metabolite may be indicative of a defect in a particular muscle metabolic pathway. Additionally, such functional tests also provide an objective measurement of exercise intolerance by comparing patient handgrip maximal MVC and cycle ergometry MP to normative values from healthy population matched for sex, age, and anthropometric data (percent-predicted values; %Pred. MVC and %Pred. MP, respectively).

Due to differences in exercise type (isometric vs. dynamic) and duration, the relative contribution of the different metabolic pathways to total ATP turnover are distinct during isometric handgrip test and cardio-pulmonary exercise test. Isometric handgrip test exercise duration is 3–5 s and is more reliant on alactic anaerobic metabolism, while aerobic metabolism is the largest contributor during a cardio-pulmonary exercise test lasting ~10 minutes^1,6^.

Furthermore, isometric handgrip test and cardio-pulmonary exercise test performances may also be affected differentially in metabolic myopathies owing to distinct exercising muscles (upper vs. lower limbs, respectively), with differences in fiber type and energy metabolism predominance^1,7^. It has long been recognized that lower limbs contain a higher percentage of slow oxidative muscle fiber than upper limbs^8^. In case of aerobic metabolism impairment, one might therefore expect a lower performance in cardio-pulmonary exercise test than in isometric handgrip test.

Accordingly, the present study aimed to evaluate the diagnostic value of percent-predicted performance reached in two exercise tests to detect metabolic myopathies among patients consulting for exercise-induced myalgia.

## Results

Eighty-three patients and thirty-five healthy controls were investigated. The patients were classified into the following five diagnostic subgroups (Table 1): McArdle disease (Glycogen storage disease type V, n = 9), respiratory chain deficiency (n = 4), complete deficiency in myoadenylate deaminase (MAD Absent, n = 10), and non-metabolic myalgia (n = 60). Control subjects and patient subgroups were well matched for sex and age (Table 1). Percent-predicted values for isometric handgrip maximal voluntary contraction force (%Pred. MVC) and maximal power during cycle ergometry (%Pred. MP) were calculated according to normative formulas given in the Materials & Methods section. %Pred. MVC was significantly lower in McArdle and non-metabolic myalgia groups (78.5 ± 9.3% and 87.6 ± 18.9%, respectively) compared to control subjects (p = 0.001 and p = 0.029, respectively; Games-Howell post-hoc test). During cycloergometer exercise, percentage of predicted maximal value for power was significantly lower in McArdle and MAD Absent patients (43.8 ± 14.6% and 71.7 ± 17.1%, respectively) compared to Controls and non-metabolic myalgia patients (p < 0.02, Post hoc Scheffe’s test). %Pred. MP was significantly decreased in Respiratory chain deficiency compared to healthy controls (67.5 ± 30.7% vs. 107.3 ± 20.9%, p = 0.016). Percentage of predicted maximal value for oxygen uptake (% Pred. peak V’O_2_) was significantly lower in McArdle patients (66.9 ± 11.1%) than in Controls and non-metabolic myalgia patients (p < 0.001, Post hoc Scheffe’s test).

**Table 1:** Anthropometric characteristics and exercise test variables in healthy controls and patients with and without metabolic myopathies.

	Control	Non-metabolic Myalgia	MAD Absent	McArdle	Respiratory Chain Deficiency
***Anthropometric data***					
Number (n)	35	60	10	9	4
Sex (f/m)	17/18	20/40	4/6	5/4	1/3
Age (years)	40.1 ± 12.9	42.5 ± 13.6	39.9 ± 15.6	43.3 ± 25.9	40.5 ± 16.4
BMI (kg.m^−2^)	23.9 ± 4.9	24.3 ± 4.5	27.1 ± 7.0	22.5 ± 3.5	24.0 ± 4.9
***Isometric handgrip test***					
MVC (DaN)	39.7 ± 8.8	38.2 ± 12.1	37.4 ± 10.8	26.7 ± 7.9 *	33.9 ± 14.1
%Pred. MVC	97.1 ± 12.4	87.6 ± 18.9^†^	87.9 ± 19.9	78.5 ± 9.3^†^	76.1 ± 30.2
***Progressive cycle ergometer test***					
Maximal power (W)	195.3 ± 64.5	183.4 ± 64.6	134.0 ± 59.7	68.1 ± 19.6^†‡§^	128.5 ± 69.5
% Pred. maximal power	107.3 ± 20.9	98.2 ± 21.9	71.7 ± 17.1 *^#^	43.8 ± 14.6 *^#^	67.5 ± 30.7 *
Peak V’O_2_ (ml.min^−1^.kg^−1^)	34.3 ± 8.2	32.6 ± 9.2	28.7 ± 12.9	21.2 ± 7.0 *^#^	27.3 ± 11.4
% Pred. peak V’O_2_	106.8 ± 21.4	100.8 ± 18.0	89.6 ± 20.3	66.9 ± 11.1 *^#^	78.7 ± 26.9
***Ratio MVC/MP***					
%Pred. MVC / %Pred. maximal power	0.92 ± 0.13	0.92 ± 0.25	1.29 ± 0.43	1.96 ± 0.66 ^†‡^	1.24 ± 0.42

Data are reported as mean ± SD. MAD, Myoadenylate deaminase; BMI, Body Mass Index; MVC, maximal voluntary contraction force; Peak V’O_2_, maximum oxygen consumption; MP, Maximal Power. Data were analyzed by a one-way ANOVA, followed by post-hoc Scheffé’s or Games-Howell pairwise comparison tests according to Levene’s test results for homogeneity of variance. ^*^significantly different from Control (p < 0.05, Post hoc Scheffe’s multiple comparison test). ^#^significantly different from non-metabolic myalgia (p < 0.05, Post hoc Scheffe’s multiple comparison test). ^†^significantly different from control (p < 0.03, Games-Howell post hoc test). ^‡^significantly different from non-metabolic myalgia (p < 0.03, Games-Howell post hoc test). ^§^significantly different from respiratory chain deficiency (p < 0.05, Games-Howell post hoc test).

We observed a non-significant decrease in % Pred. peak V’O_2_ in Respiratory chain deficiency compared to control subjects (78.7 ± 26.9% vs. 106.8 ± 21.4%, p = 0.112).

**Figure 1:**
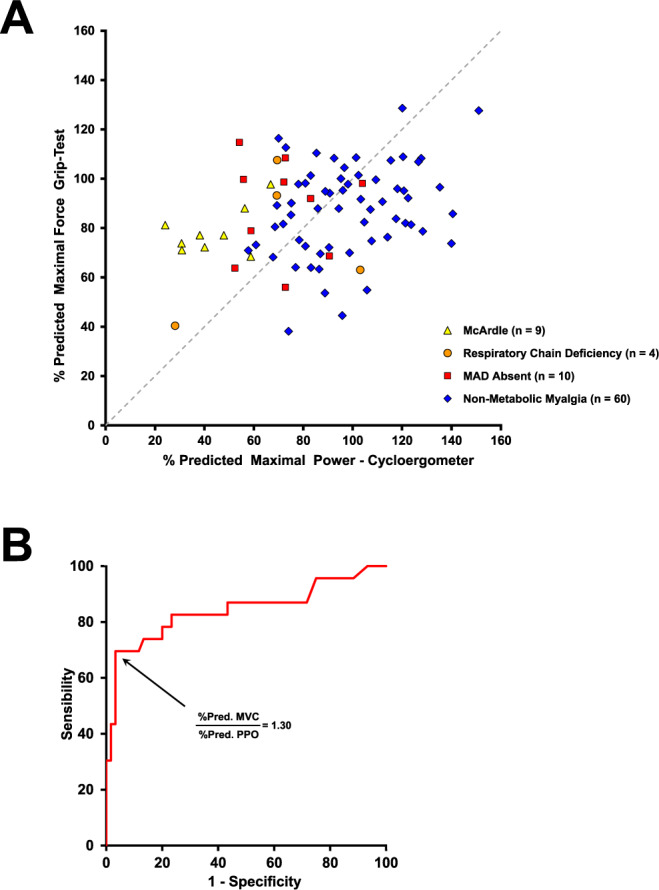
Predicted maximal handgrip force vs. predicted maximal double-leg cycling power in patients consulting for exercise intolerance and exercise-induced myalgia. (**A**) The % predicted handgrip MVC is plotted against the % predicted maximal power (MP) during cardio-pulmonary exercise test. The grey dashed line represents equal % predicted values for handgrip MVC and MP (iso-line). (**B**) Receiver operating characteristic (ROC) curve to distinguish metabolic myopathies from non-metabolic myalgia using MVC%pred:MP%pred ratio as predictor variable. The area under the ROC curve (0.843; 0.758–0.927, 95% CI) indicates the probability that a randomly selected pair of metabolic myopathy and non-metabolic myalgia patients will be accurately classified as to their disease state. The optimal cut-off for MVC%pred:MP%pred ratio was selected as 1.30 to discriminate metabolic myopathies from non-metabolic myalgia.

A scatter plot presenting the %pred. MVC in handgrip test vs. %Pred. maximal power in cycloergometry in patients is shown in Fig. 1A. The dashed line represents values where %pred. MVC and %Pred. maximal power in cycloergometry are equal (‘iso-line’), and delineates two distinct profiles of exercise performance. Subjects above the line (upper-left zone) exhibit a higher %Pred. value for handgrip MVC compared to %Pred. maximal power in cycloergometry. In contrast, patients below the line (lower-right zone) exhibit a higher %Pred. maximal power in cycloergometry compared to %Pred. value for handgrip MVC.

A visual inspection of the plots in Fig. 1A suggests distinctive profiles for metabolic myopathies and non-metabolic myalgia. Metabolic myopathies are mainly located in the upper-left zone, whereas non-metabolic myalgias are mainly located in the lower-right zone.

To further assess the different patterns, we calculated for each subject the ratio between the percentage of predicted value reached for handgrip MVC and maximal power in cycloergometry (Table 1). The MVC%pred:MP%pred ratio was significantly higher in McArdle (1.96 ± 0.66) compared to control (0.92 ± 0.13) and non-metabolic myalgia (0.92 ± 0.25) groups (p = 0.009 and p = 0.009, respectively; Games-Howell post-hoc test). When considered as a single group, patients with metabolic myopathies displayed a higher MVC%pred:MP%pred ratio (1.54 ± 0.62) than non-metabolic myalgia and Controls (p < 0.001, Games-Howell post-hoc test).

A receiver operating characteristic (ROC) curve was generated to evaluate the ability of MVC%pred:MP%pred ratio to predict metabolic myopathy in patients consulting for exercise-intolerance and exercise-induced myalgia (Fig. 1B). ROC analysis showed that the area under the curve (AUC) was 0.843 (0.758–0.927, 95% CI). A MVC%pred:MP%pred ratio of 1.30 was selected as the optimal cutoff because it provided the highest sum of sensitivity and specificity minus one (Youden’s index = 0.66, Se = 69.6% and Sp = 96.7%). The positive and negative likelihood ratios (LR + and LR−) of this cutoff were 20.9 (5.2–83.7, 95% CI) and 0.32 (0.17–0.59, 95% CI), respectively. The overall diagnostic accuracy of a 1.30 cutoff for MVC%pred:MP%pred ratio was 66.3 (12.5–350.7, 95% CI).

**Figure 2:**
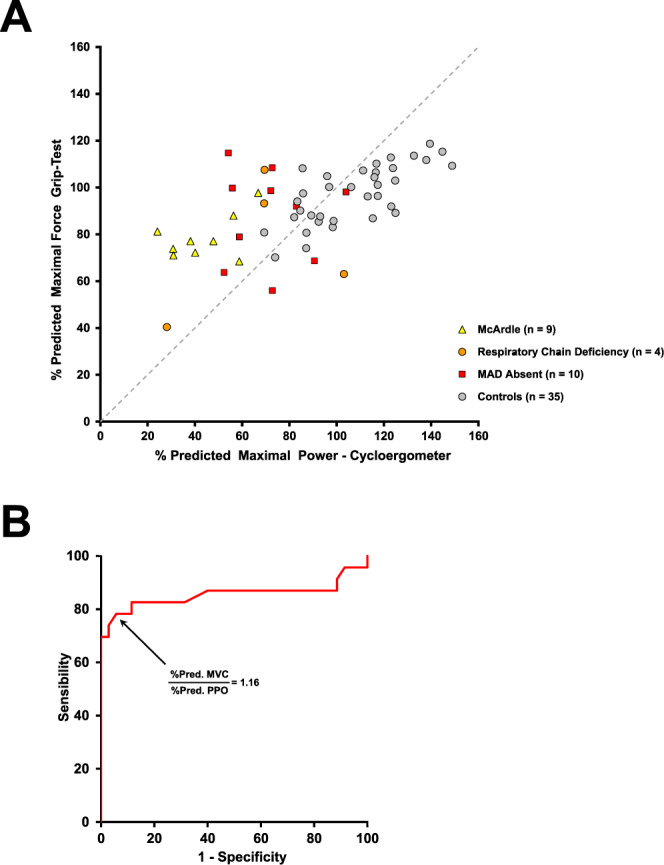
Predicted maximal handgrip force vs. predicted maximal double-leg cycling power in metabolic myopathies and healthy controls. (**A**) The % predicted handgrip MVC is plotted against the % predicted maximal power (MP) during cardio-pulmonary exercise test. The grey dashed line represents equal % predicted values for handgrip MVC and MP (iso-line). (**B**) Receiver operating characteristic (ROC) curve of MVC%pred:MP%pred ratio for differentiating metabolic myopathies from healthy controls. The area under the ROC curve is 0.855 (0.760–0.951, 95% CI). The optimal MVC%pred:MP%pred ratio was 1.16 to discriminate metabolic myopathies from healthy controls.

The discriminatory ability of MVC%pred:MP%pred ratio to differentiate metabolic myopathies from control subjects was also evaluated (Fig. 2). The ROC curve AUC for MVC%pred:MP%pred ratio was 0.855 (0.760–0.951, 95% CI) for distinguishing between control subjects and metabolic myopathies (Fig. 2B). The best cutoff point is 1.16 for MVC%pred:MP%pred ratio with 78.3% sensitivity, 94.6% specificity, and a diagnostic odds ratio of 59.4 (10.5–337.6, 95% CI).

## Discussion

The present results provide new insights into metabolic myopathies, from both pathophysiological and diagnostic perspectives. Comparison of isometric handgrip MVC and maximal power on cycloergometer appears to be useful in distinguishing patients with defects of muscle metabolism from those with non-metabolic myalgia. Interestingly, metabolic myopathies and non-metabolic myalgia display different patterns in handgrip-test and cycloergometer exercise. In metabolic myopathy patients, cycling maximal power is more impaired than handgrip MVC. The physiological bases of these two functional tests are different. As a short-term maximal exercise, handgrip MVC represents the ability to generate maximum cross-bridges within forearm and depends on pre-exercise ATP store^1,6^. In contrast, aerobic metabolism meets most of the high ATP turnover rate during cardio-pulmonary exercise test^1,6^. Accordingly, it seems obvious that an impairment of intermediary metabolism, as occurring during metabolic myopathies^9^, will impact heavily the maximal -aerobic- power in cycloergometry.

As muscle weakness and exercise-induced myalgia are symptoms frequently encountered in clinical practice, a detailed questioning and a careful clinical examination of the patient are mandatory before considering a primary muscle disease^10,11^. Therefore, the diagnostic evaluation of patients presenting with exertional myalgia is usually labor intensive and requires ancillary specialized tests, such as electromyogram, muscle biopsy, and genetic testing^10,11^. The clinical approach to the patient suspected of metabolic myopathy commonly uses exercise testings^5,9,12^, but few studies have discussed in which sequence isometric handgrip test and cardio-pulmonary exercise test should be performed in the diagnostic work-up^12^. The current study strongly suggests that both tests should be performed, and their peak mechanical performances combined in a composite index. The MVC%pred:MP%pred ratio may help to identify metabolic myopathies among patients consulting for exercise intolerance and direct genetic testing or specific histochemical analysis on muscle biopsies.

For a MVC%pred:MP%pred ratio cut-off value of 1.30, the corresponding positive and negative likelihood ratios were 20.9 and 0.32, respectively. The positive likelihood ratio is probability of an individual with the condition having a positive test. Accordingly, LR + and LR− are proportions of patients who are correctly judged as positive or negative by a diagnostic test^13^. According to Mcgee^14^, a LR + > 10 represents a ‘large’ increase in the probability of disease when the test is positive, while a LR− in the range of 0.1–0.2 represents a ‘moderate’ decrease of probability when the test is negative. A LR + at 20.9 indicates that a patient with a metabolic myopathy is about 20.9 times more likely to have a positive test than a subject with non-metabolic myalgia. By using conversion to an odds ratio, the LR modifies the pretest probability of a given diagnostic test for a suspected disorder. According to Bayes’ theorem, the pretest odds of metabolic myopathies multiplied by the likelihood ratio gives the posttest odds of metabolic myopathies. In our unit, subjects with metabolic myopathies represent 15% of referred patients for metabolic exercise testing (pretest probability). Thus, the prestest odds for metabolic myopathy (probability × 1 – probability = 0.15/1–0.15) is 0.18. The posttest odds can be calculated by multiplying the pretest odds by LR (0.18 × 20.9 = 3.68). Finally, the posttest probability for metabolic myopathy is 78.6% (odds/1 + odds = 3.68/1 + 3.68 = 0.786, or 78.6%). Interestingly, since diagnostic tests are commonly conducted in sequence, the posttest odds of exercise tests becomes the pretest odds for genetic testing or muscle biopsy in the diagnostic work-up of exercise-induced myalgia.

An advantage of MVC%pred:MP%pred ratio is avoidance of blood drawing to determine muscle metabolite concentration. However, we acknowledge that the present index is designed as a screening method to rule in/out a metabolic myopathy, and not to provide a diagnosis for a given metabolic myopathy. If a specific diagnosis is searched, such as mitochondrial myopathy, more specific functional tests that include blood metabolite (i.e., lactate, ammonia) and venous oxygen measurements should be performed^2,15,16^. Another potential limitation is that maximal isometric handgrip force is highly dependent on central motor drive (‘voluntary activation’). Vigorous and standardized verbal encouragement during MVC collection trials is needed to maximize both the number of active motor units and measurement reproducibility.

In conclusion, the MVC%pred:MP%pred ratio appears helpful in the detection of metabolic myopathies by altering the pretest probability substantially. As a result, the diagnostic work-up will be more cost-effective and less time-consuming.

## Materials and Methods

All testing procedures were approved by the local research ethics of Brest Universitary Hospital and conformed to the principles set by the Declaration of Helsinki (Clinical Trial NCT02362685). Subjects gave written informed consent to participate in the present study. Participants were requested to refrain from exercise and alcohol consumption in the 24 hours before the tests. This diagnostic study was performed in compliance with the Standards for Reporting of Diagnostic Accuracy (STARD) recommendation. Eighty-three patients consulting for exercise intolerance and myalgia in Brest neuromuscular center (University Hospital, Brittany, France) were included.

### Subjects

The different subgroups of patients are reported in Table 1. In McArdle group (n = 9), disease was diagnosed by myophosphorylase absence in muscle biopsy (n = 6, Supplementary Fig. S1 online)^17^, and by documented mutations in *PYGM* gene in all patients (c.148 C > T/c.148 C > T, n = 7; c.148 C > T/c.2262delA, n = 1; c.148 C > T/c.1466 C > G, n = 1). *PYGM* gene (20 exons and intron-exon junctions) was sequenced from PCR-amplified genomic DNA and compared to reference sequence (NM_005609.2).

Respiratory chain deficiency group (n = 4) consisted of one patient with MELAS syndrome (m.3243 A > G *MTTL1* gene mutation, muscle tissue heteroplasmy: 55%), two patients with chronic progressive external ophthalmoplegia (40 and 30% cox-negative fibers at muscle biopsy, Supplementary Fig. S1 online; 4 and 6 kb mtDNA deletions, respectively), and one patient displaying 10% cox-negative fibers at muscle biopsy with decreased complex IV activity (40% of normal, spectrophotometric enzyme assay).

Deficiency in MAD activity was assessed by histochemical analysis of 10 µm muscle sections stained with p-nitro blue tetrazolium (Supplementary Fig. S1 online), as previously reported^4,5,18,19^. In ten patients, histochemical reaction for MAD was absent. In this group (MAD Absent), analysis of the *AMPD1* gene revealed that nine individuals were homozygous for the c.133 C > T mutation and one was compound heterozygote (c.133 C > T/c.567 G > T). The ten patients with absent MAD activity had no evidence of other associated myopathy after their diagnostic work-up (see below).

Non-metabolic myalgia group included sixty patients presenting exercise intolerance with exercise-induced myalgia, and no final diagnosis despite an exhaustive evaluation. For all these subjects, the diagnostic work-up included clinical examination, routine lab test, acylcarnitine profile, electromyography, no abnormality identified in a muscle biopsy analyzed according to standardized national guidelines^20^, and discussion at multidisciplinary team meeting.

Thirty-five healthy individuals, who performed both the grip-test and the cardio-pulmonary exercise testing under the same conditions and within the same recruitment period as patients, were included as a Control group.

### Grip-test

Maximal handgrip force was measured in dominant arm by a strain gauge torsion dynamometer (MIE Medical Research Limited, Leeds, UK) in subjects placed in a semi-seated position^2,3^. The test procedures were standardised using a specific software to monitor protocol timing^2,3^, and data collection was carried out by the same tester. Subjects were familiarized with the dynamometer and instructed to squeeze the instrument as hard as possible. Subjects performed three maximal voluntary contraction (MVC; 3–5 sec) interspersed with 1-min rest periods^2,3^. The within subject coefficient of variation for the three MVC measurements (quotient of the standard deviation and the mean value) was 4.8 ± 2.9%. The maximum value among the three measurements was used as maximal voluntary contraction force (MVC)^2,3^, and expressed as percentage of predicted value^3^: MVC_Predicted_ (DaN) = (2.699 × forearm diameter_cm_) + (5.979 × sex_male=1, female=0_) − (0.244 × age_yr_) − 22.776.

### Bike

Subjects underwent a progressive, power-incremented cycling test on a magnetically braked cycle ergometer (Ergoline GmbH, Bitz, Germany) to determine the mechanical power eliciting the individual-specific maximum rate of oxygen uptake. Increasing workloads were applied using a stepwise protocol, which was adjusted for each individual in order to achieve maximum exertion within ten minutes. Age- and height-predicted value for maximal power was calculated according to Jones equations^21^: for male, maximal power = 245.48 × height_cm_^2.7^ × age_yr_^−0.46^, and for female, maximal power = 157.95 × height_cm_^2.8^ × age_yr_^−0.43^. This theoretical maximal power was adjusted according to exercise intolerance (Bader)^22^ in order to obtain an estimated maximal power (EMP). After a 2-min warm-up at the intensity of 20%EMP with a pedalling cadence of 60 rpm, work rate was increased by 10%EMP.min^−1^ until volitional exhaustion (i.e., inability to sustain the required pedalling rate of 60 rpm). Ventilation and pulmonary gas exchange were measured breath-by-breath at rest and during exercise using an open circuit system (Medical Graphics Corporation, St. Paul, Minnesota). Each subject was monitored during cardio-pulmonary exercise test by continuous 12-lead ECG-telemetry and blood pressure^4,5^. The maximum rate of oxygen uptake (V’O_2max_) was expressed relative to bodyweight (mL/min/kg) and as a percent-predicted value^23^. Maximal power was expressed as a percentage of the predicted normal value given by Jones equations.^21^

### Statistics

Statistical differences between groups were tested using ANOVA with a post hoc test if a significant main effect was found. Scheffe and Games-Howell post hoc tests were used in cases of equal and unequal variances of data (Levene’s test), respectively.

The diagnostic performance of MVC%pred:MP%pred ratio to discriminate metabolic myopathy from non-metabolic myalgia and control subjects was assessed by ROC curve analyses^4,5,24^. The MVC%pred:MP%pred value at which the Youden index (sensitivity + specificity – 1) is maximal was considered the optimal cutoff^24^. The positive likelihood ratio [LR + = sensitivity/(1 − specificity)], the negative likelihood ratio [LR− = (1 − sensitivity)/specificity], and the diagnostic odds ratio (LR + divided by LR−) corresponding to this cutoff were calculated^13^. Data are reported as mean ± SD. p values under 0.05 were considered significant. Statistical analysis was conducted using SPSS Statistics 25 (SPSS, Inc, Chicago, Illinois, USA).

## Supplementary information

Supplementary information.

## Data Availability

The datasets generated during and/or analyzed during the current study are available from the corresponding author upon reasonable request.
