# Supplementary material for: The ratio of maximal handgrip force and maximal cycloergometry power as a diagnostic tool to screen for metabolic myopathies
Source: Sci Rep. 2020 Jun 1;10:8865. doi: 10.1038/s41598-020-65797-1 (PMC7264313; doi:10.1038/s41598-020-65797-1)

Title : The ratio of maximal handgrip force and maximal cycloergometry power as a diagnostic tool to screen for metabolic myopathies

Authors: Jean-Baptiste Noury, Fabien Zagnoli, François Petit, Cédric Le Maréchal, Pascale Marcorelles, Fabrice Rannou

Supplementary materials legend:

S1. Histoenzymological techniques

Histoenzymology was performed on serial transverse cryostat sections of muscle samples (10 µm thick) obtained using the open biopsy technique.

Upper panels: Myophosphorylase reaction performed as previously described.[17] (**A**), Normal myophosphorylase staining (non-metabolic myalgia patient) with a checkerboard pattern of purple-stained fibers, type 2 fibers showing more intense staining. (**B**), Absence of phosphorylase staining in muscle fibers (McArdle patient); note that some residual phosphorylase staining is present in intramuscular blood vessel walls.

Middle panels: Cytochrome c oxidase-succinate dehydrogenase (COX-SDH) overlap staining performed according to Dubowitz *et al.* (2013).[17] (**C**), Normal COX-SDH staining (non-metabolic myalgia patient). (**D**), Biopsy shows fibers manifesting mitochondrial accumulation and focal COX-deficiency (patient with chronic progressive external ophtalmoplegia).

Lower panels: Myoadenylate deaminase (MAD) staining with *p*-nitro blue tetrazolium according to Fishbein *et al.* (1980).[17,18] (**E**), Normal MAD activity (non-metabolic myalgia patient). (**F**), Absent MAD activity. Scale bar, 50 µm.

Fig. S1

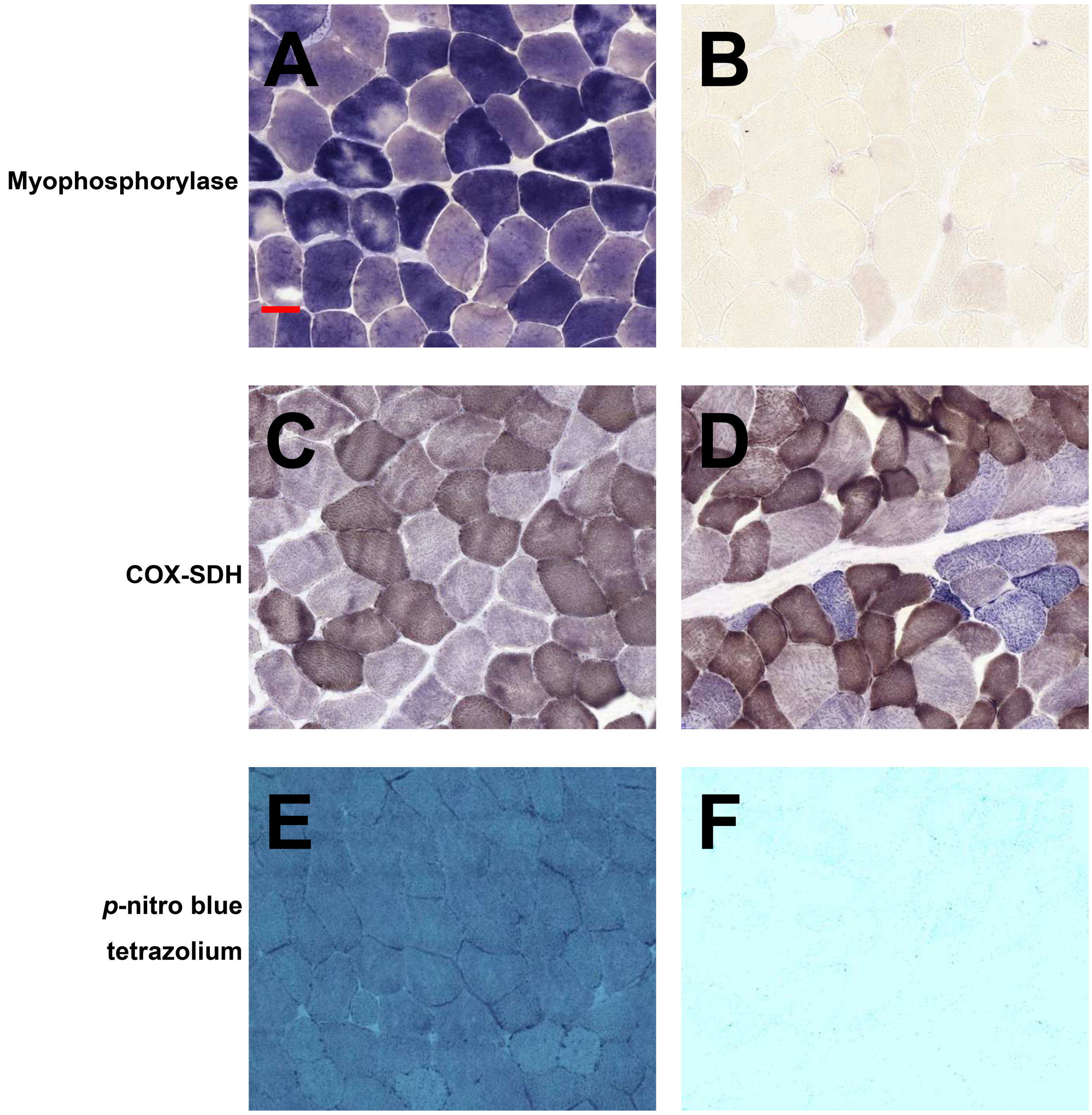

Supplement: Supplementary file 1 — Supplementary information. [file 41598_2020_65797_MOESM1_ESM.pdf]
